# Analysis of lncRNAs Expression Profiles in Hair Follicle of Hu Sheep Lambskin

**DOI:** 10.3390/ani10061035

**Published:** 2020-06-15

**Authors:** Xiaoyang Lv, Weihao Chen, Wei Sun, Zahid Hussain, Shanhe Wang, Jinyu Wang

**Affiliations:** 1 College of Animal Science and Technology, Yangzhou University, Yangzhou 225009, China; dx120170085@yzu.edu.cn (X.L.); 18552133709@163.com (W.C.); zahidbiochem737@gmail.com (Z.H.); shanhe12315@163.com (S.W.); 2 Joint International Research Laboratory of Agriculture and Agri-Product Safety of Ministry of Education of China, Yangzhou University, Yangzhou 225009, China; 3 Jiangsu Co-Innovation Center for Important Animal Infectious Diseases and Zoonoses, Yangzhou University, Yangzhou 225009, China

**Keywords:** Hu sheep, lambskin, hair follicle, lncRNAs

## Abstract

**Simple Summary:**

Lambskin trait is one of the important economic traits of Hu sheep, and its quality depends on the pattern type of lambskin, which is affected by hair follicle growth and development. In our study, RNA-seq was used to detect the expression profiles of long non-coding RNAs (lncRNAs) between straight wool and small waves of the hair follicle in Hu sheep lambskin. The results showed that a total of 75 differentially expressed lncRNAs, and 25 differentially expressed mRNAs were screened between small waves group and straight wool group. Functional annotation of differentially expressed mRNAs showed that *FGF12* and *ATP1B4* were enriched in MAPK, PI3K-Akt, and Ras signaling pathways, which had a certain influence on hair follicle growth and development. Interestingly, TCONS_00279168 was predicted to target *FGF12* and its source gene *ATP1B4.* Thus, TCONS_00279168, *FGF12,* and *ATP1B4* can be used for follow-up research and provide a new idea for the molecular mechanism in Hu sheep hair follicle growth and development.

**Abstract:**

Lambskin of the Hu sheep exhibits high economic value due to its water-wave pattern. Wool curvature is the key factor of the pattern types and quality of lambskin, and it is formed by the interaction between dermal papilla cells and hair matrix cells in the hair follicle, which is regulated by various genes and signaling pathways. Herein, three full-sibling pairs of two-day-old healthy lambs (*n* = 6) were divided into a straight wool group (ST) and small waves group (SM) with three repetitions. RNA-seq was applied to determine the expression profile of mRNAs and lncRNAs in Hu sheep hair follicles. 25 differentially expressed mRNAs and 75 differentially expressed lncRNAs were found between SM and ST. FGF12, ATP1B4, and TCONS_00279168 were probably associated with hair follicle development. Then, Gene Ontology (GO) and KEGG enrichment analysis were implemented for the functional annotation of target genes of differentially expressed lncRNAs. The results showed that many genes, such as *FGF12* and *ATP1B4,* were found enriched in PI3K-Akt signaling, MAPK signaling, and Ras signaling pathway associated with hair follicle growth and development. In addition, the interaction network of differentially expressed lncRNAs and mRNAs showed that a total of 6 differentially expressed lncRNAs were associated with 12 differentially expressed mRNAs, which may be as candidate mRNAs and lncRNAs. TCONS_00279168 may target *ATP1B4* and *FGF12* to regulate MAPK, PI3K-Akt, Ras signaling pathways involved in the sheep hair follicle development process. These results will provide the basis for exploring hair follicle development.

## 1. Introduction

Hu sheep, as an indigenous sheep breed in China, is world-wild famous for its water-wave lambskin [1]. The width of the pattern and the bending degree of the wool is the most important indicators to evaluate the quality of Hu sheep lambskins [2]. According to the indicators, the lambskin of Hu sheep can be divided into four levels: Small waves, medium waves, large waves, and straight wool, in which small waves have the best quality while the straight wool is the worst [3]. In fact, the formation of the different pattern mainly depends on the degree of wool curvature, which is closely associated with the hair follicle growth and development. The growth and interaction of various cells in the hair follicle, such as dermal papilla cells, hair matrix cells, outer root sheath cells, and inner root sheath cells, can contribute to asymmetrical growth on two sides of wool, which in turn causes the wool curvature [4]. Thus, the hair follicle is the focus to explore wool curvature.

The molecular mechanism of lambskin trait has been a research hotspot since the 1980s. Huge evidence [5,6,7] has theoretically highlighted the role of hair follicle morphogenesis in lambskin trait. Hair is a kind of fiber produced by the hair follicle, length, and fineness of the hair, which causes wool curvature. The formation of wool curvature is associated with symmetry/asymmetry relationships of hair follicles, which can be traced back to hair follicle growth and development. To date, several major pathways have already been proved to show symmetry/asymmetry formation, including Wnt signaling pathway [8,9], TGF-β signaling pathway [10] and BMP signaling pathway [11] and others, are hair follicle growth and development, as well as the formation of wool curvature. BMPs (*BMP4*, *BMP7)* [12,13], FGFs (*FGF20, FGF10, FGF4*) [4,14,15], *Sox2*, *Sox18* [16,17] are closely related to hair follicle development and wool curvature.

Furthermore, with the rapid development of RNA sequencing techniques, the interest in non-coding RNAs (ncRNA) has increased. ncRNAs were found to have various important biological roles in mammals such as diseases, reproduction, and hair follicle development [18,19]. Considerable researches in non-coding RNAs have examined lncRNAs, which play a vital role in transcriptional regulation [20]. Although some lncRNAs have been reported to be involved in the hair follicle, the role of the lncRNAs in wool curvature is unclear. In recent years, Yue et al. discovered 15 significant differentially expressed lncRNAs. Among them, XLOC005698 was predicted to bind competitively to oar-miR-3955-5p to regulate hair follicle development [21]. Beyond that, lncRNA PlncRNA-1 was validated that it could participate in the biological regulatory process of proliferation and differentiation of hair follicle stem cells by regulating TGF-β1 to lead to the biological changes in Wnt/β-catenin signal pathway [22]. No researches have linked hair follicle development to wool curvature. The formation of lambskin pattern in Hu sheep, depending on wool curvature, is also influenced by hair follicle development, mainly the interaction between dermal papilla cells and hair matrix cells in the hair follicle. Taken into consideration, it is a great direction to explore the regulation mechanism of lncRNA on hair follicle development, especially the growth and development of dermal papilla cells and hair matrix cells.

In this study, to screen differentially expressed lncRNAs and mRNAs between straight wool group (ST) and small waves group (SM) for the preparations of exploring the mechanism of lncRNA regulating hair follicle development, Hu sheep with straight wool group and small waves group were selected and RNA-Seq was applied for the identification of expression profiles of lncRNAs. Furthermore, the systematic analysis was performed to investigate the potential roles of lncRNAs. Our study may pave the way for an in-depth study of the molecular mechanism in sheep lambskin trait.

## 2. Material and Methods

### 2.1. Animals and Samples

In order to minimize the pain of animals during the experiment, all experimental procedures mentioned in the present study strictly followed the requirement with the Jiangsu Provincial Government Animal Care and Use Committee (IACUC) (License Number: 45) and the Chinese Ministry of Agriculture (License Number: 39). All experimental procedures were approved by the Animal Care and Use Committee at Yangzhou University.

Two-day-old healthy Hu lambs (*n* = 6) were collected in Suzhou Stud Farm in Jiangsu Province, China. A total of 3 pairs of full-sib individuals were divided into straight wool groups and small waves group. Around 1 cm of hair root from the dorsal side of the Hu lambs was cut off and collected in the freezing tube with Drikold until about a 1/3 volume. All samples were snap-frozen in liquid nitrogen and then placed at −80 °C for RNA extraction.

### 2.2. Library Construction and Sequencing

The experimental procedures of the RNA library construction were strictly carried out under the manufacturer’s instructions. Total RNA was extracted from the hair follicle tissues. RNA purity and concentration were immediately checked using the Nanodrop (Thermo Fisher, Shanghai, China). RNA degradation and contamination were monitored on 1% agarose gels. RNA integrity was checked by Agilent 2100 bioanalyzer (Agilent Technologies, Santa Clara, CA, USA) and the RIN was greater than 8.0. Then, 3 µg total RNA of each sample was collected to construct the RNA library. Firstly, ribosomal RNA was removed using the Epicentre Ribo-zero™ rRNA Removal Kit (Epicentre, Madison, WI, USA), and the rRNA was cleaned up by ethanol precipitation. Subsequently, the first strand cDNA was synthesized by a random primer, and the second strand cDNA was subsequently synthesized by DNA Polymerase I. AMPure XP system (Beckman Coulter, Brea, FL, USA) was used to select the cDNA fragments (150–200 bp). Next, the cDNA library was assessed by Agilent Bioanalyzer 2100 system. After that, these cDNA libraries were sequenced on Illumina PE 150 system (Pair end 150 bp). 

### 2.3. Quality Control of Raw reads and Genome Alignment

In order to ensure the accuracy of subsequent analysis, quality control of the raw read was carried out. Raw reads were filtered by removing the read with ploy-N, adapter, and low quality. Moreover, the error rate and GC content were estimated for subsequent analysis. At the same time, clean reads were mapped to the *Ovis aries* reference genome (Oar_v3.1) by STAR (v2.5.1b). 

### 2.4. Identification of lncRNAs

According to the structural and functional characteristics of lncRNA, a series of strict screening conditions were conducted through the following steps. Firstly, the single exon transcripts with low reliability were filtered, and transcripts with exon number ≥ 2 were retained. Secondly, transcripts with a length > 200 nt were selected. Thirdly, transcripts the same or similar to the transcripts of pre-miRNA, mRNA, tRNA, snoRNA, snRNA, and rRNA were removed using Cuffmerge software. Then, CPC [23], CNCI [24], Pfam [25], and PLEK [26] were used to predict the coding potential of lncRNA transcripts, and transcripts with no coding potential were selected. 

### 2.5. Quantification and Differential Expression Analysis

The fragments per kilobase per million mapped reads (FPKM) method [27] was applied to estimate the expression levels of the lncRNA and mRNA transcripts. EdgeR package [28] was used to discover the differentially expressed lncRNAs between straight (ST) and small waves (SM) with 3 biological replicates. Furthermore, *p*-values were adjusted by Benjamini and Hochberg’s approach and corrected *p*-value < 0.05 was assigned as statistically significant.

### 2.6. Prediction and Enrichment Analysis of lncRNA Target Genes

The co-location and co-expression analysis methods were used to predict the target genes of lncRNAs. In *cis* regulation, the coding genes located in around 100K upstream and downstream of lncRNAs were considered the target genes, and targets in *trans* regulation were predicted based on the condition that the correlation coefficient was greater than 0.95.

Gene Ontology (GO) (http://www.geneontology.org/) and KEGG (http://www.genome.jp/kegg/) pathway analysis were implemented by the hypergeometric test method, and adjusted *p*-value < 0.05 was considered significant by Fisher’s test. The clusterProfiler R package was carried out to test the statistical enrichment of differentially expressed genes in GO and KEGG analysis [29].

### 2.7. Interaction Relationship Analysis of Differentially Expressed lncRNAs and mRNAs

For a better understanding of the function of lncRNAs, the interaction relationship analysis of differentially expressed lncRNAs and mRNAs was carried out. Not only the position correlation but also the expression correlation was considered, and then, the interaction relationship was constructed by Cytoscape software [30].

### 2.8. Data Validation

The RNAs from the hair follicle of Hu sheep used for RNA-seq were simultaneously used for RT-qPCR analysis. The first-strand cDNA synthesized by PrimeScriptTM Reagent Kit gDNA Eraser (Takara, Dalian, China). Differentially expressed lncRNAs (*n* = 5) and mRNAs (*n* = 5) were randomly selected (Table 1) to verify the accuracy of RNA-seq using RT-qPCR with GAPDH as a reference gene [21,31]. Primers were designed using the Premier 5.0 software (Premier Biosoft, San Francisco, CA, USA) (Table S1). 

RT-qPCR amplification was performed in triplicate wells according to the following conditions: Initial denaturation at 95 °C for 5 min, followed by 40 cycles of 95 °C for 10 s, and 60 °C for 30 s. The melting temperature peak at 85 °C ± 0.8 on the dissociation curve determined the specificity of PCR amplification. The RT-qPCR was performed in CFX96 Touch™ (Bio-Rad, California, CA, USA).

The 2^−ΔΔCt^ method [32] was used to process the real-time PCR results. Statistical analysis was performed through SPSS 19.0 software (SPSS Inc., Chicago, IL, USA). The levels of gene expression were analyzed for significant differences with a dependent sample t-test. All experimental results were shown as mean ± SEM. A probability of *p* ≤ 0.05 was considered statistically significant.

## 3. Results

### 3.1. Overview of Sequencing Results

On average, 110,304,537 (SM) and 98,836,421 (ST) raw reads were obtained from the hair follicle. The average number of clean reads in SM and ST were 108,481,996 and 96,677,361, with the GC contents of 51.67% and 53.33%, respectively. Nearly 83.92% and 83.98% of clean reads were mapped to the *Ovis aries* reference genome, among of which uniquely mapped was more than 74.09%, on average, respectively (Table 2). 

**Figure 1:**
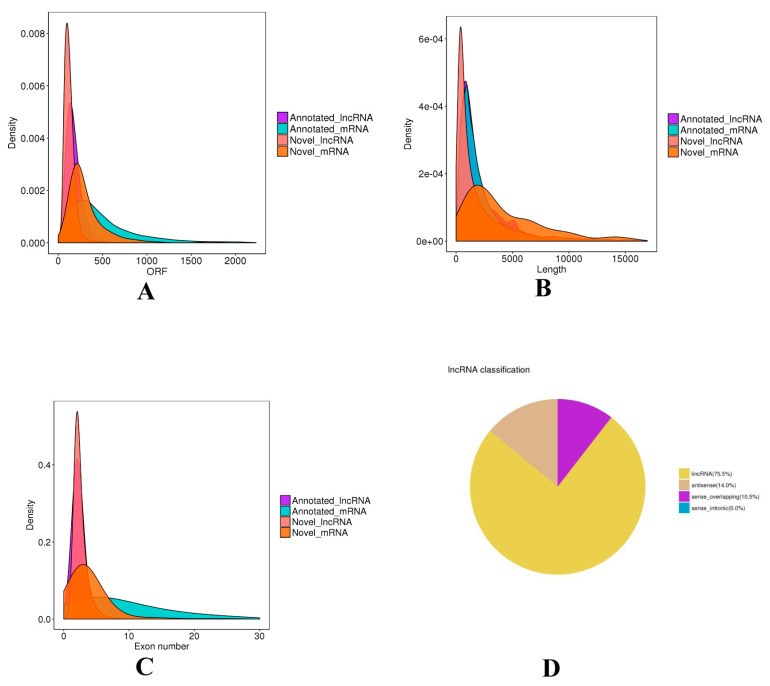
The classification and characteristic of lncRNAs. (**A**) The open reading frame (ORF) length of lncRNAs and mRNAs. (**B**) The length of lncRNAs and mRNAs. (**C**) The exon number of lncRNAs and mRNAs. (**D**) The classification of lncRNAs.

Subsequently, in total 9954 lncRNAs were identified after the coding potential filter. Furthermore, a total of 17,786 mRNAs were obtained. The ORF length, transcript length, and exon number of mRNAs and lncRNAs were calculated and compared (Figure 1). The ORF length of lncRNAs was concentrated mainly from 30 to 240 nt, and significantly shorter than that of mRNAs. The average length of lncRNAs was 2450 nt, and most of the lncRNAs were concentrated in the length range of 200 to 1000 nt, whereas that of mRNAs was longer. (Figure 1A). The majority of lncRNAs have 2-3 exons, while the exon number of mRNAs was greatly different with a range of 2 to 30. (Figure 1B). Moreover, we found that lncRNAs were mainly divided into intergenic lncRNAs (75.5%), antisense (14.0%), sense_overlapping (10.5%) (Figure 1D). 

### 3.2. Differentially Expressed Analysis

**Figure 2:**
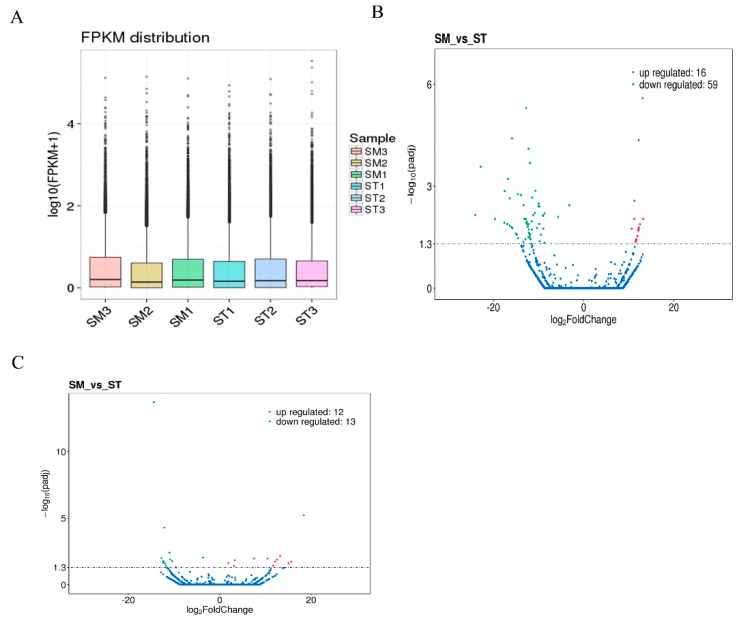
Screening and clustering analysis of differentially expressed lncRNAs and mRNAs. (**A**) Distribution of lncRNAs expression level. The expression was normalized with fragments per kilobase per million mapped reads (FPKM). (**B**) The volcano plot distribution of differentially expressed lncRNAs. (**C**) The volcano plot distribution of differentially expressed mRNAs.

The FPKM method was used to estimate the expression levels of all transcripts in SM and ST (Figure 2A). Then, significant analysis was performed with ST as a reference sample. Regarding differentially expressed lncRNAs, a total of 75 with 16 upregulated lncRNAs and 59 downregulated lncRNAs (Figure 2B) were found between SM and ST, regarding differentially expressed mRNAs (Table S2), a total of 25 with 12 upregulated mRNAs and 13 downregulated mRNAs (Figure 2C, Table S3) were found between SM and ST, under conditions of |log2 (fold change)| > 1 and corrected *p*-value threshold < 0.05.

**Figure 3:**
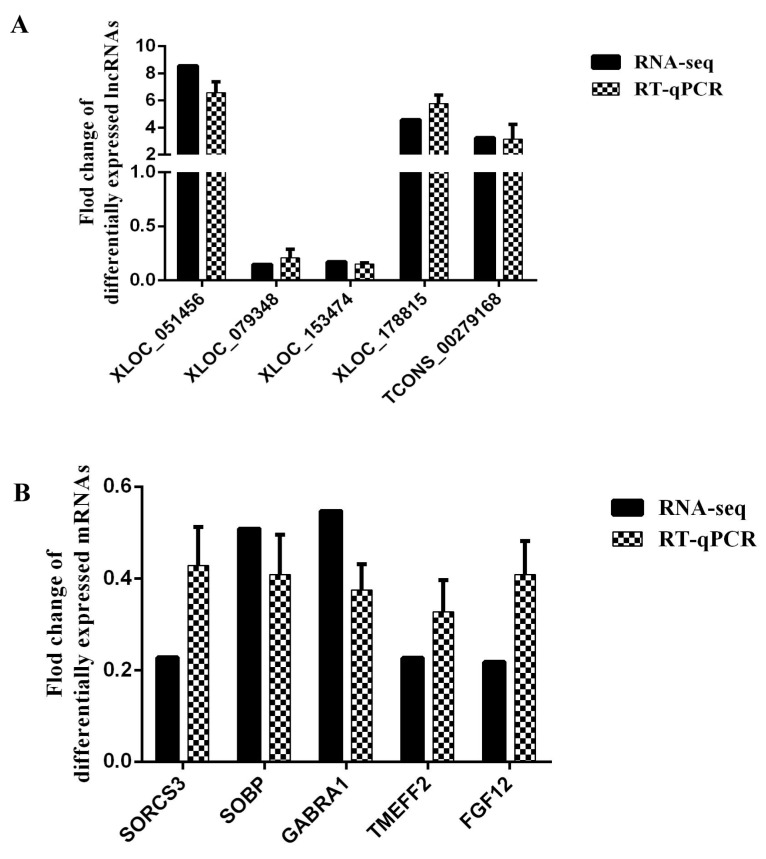
Differentially expressed lncRNAs and mRNAs from RNA-seq were verified using RT-qPCR. (**A**) The relative expression level of differentially expressed lncRNAs (*n* = 5) based on straight wool as a control group. (**B**) The relative expression level of differentially expressed mRNAs (*n* = 5) based on straight wool as a control group.

The validation results for the randomly selected differentially expressed lncRNAs and differentially expressed mRNAs indicated that the results were similar to the RNA-seq, suggesting that the results of RNA-seq were reliable (Figure 3).

### 3.3. Functional Enrichment Analysis of Differentially Expressed lncRNAs

**Figure 4:**
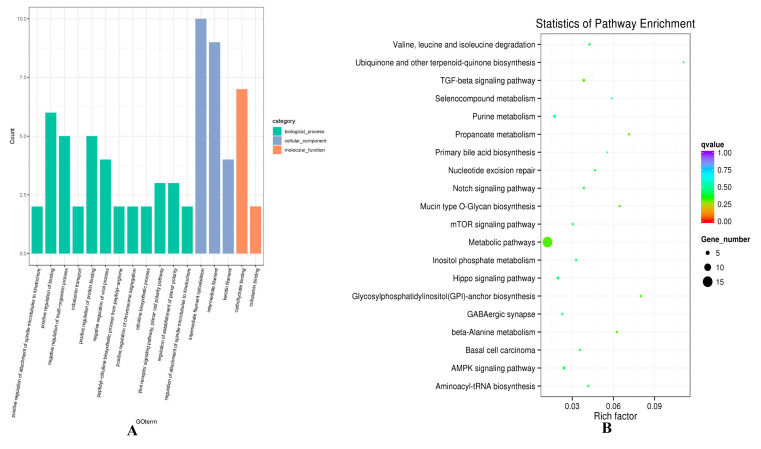
Gene ontology (GO) and Kyoto Encyclopedia of Genes and Genomes (KEGG) analysis of differentially expressed lncRNAs target mRNA through co-location analysis in hair follicle. (**A**) The enrichment GO terms between small waves and straight wool. (**B**) The enrichment pathways between small waves and straight wool.

Considering the mechanism of regulating gene expression, the potential target genes of 75 differentially expressed lncRNAs were predicted, including co-location and co-expression analysis (Table S4, Table S5). Functional enrichment analysis was conducted to predict biological function. Regarding the co-location result, GO function analysis showed that GO items were enriched for intermediate filament cytoskeleton (GO:0045111), intermediate filament (GO:0005882), keratin filament (GO:0045095), and Wnt receptor signaling pathway, planar cell polarity pathway (GO:0060071) (Figure 4A, Table S6). KEGG enrichment analysis revealed that the target genes of differentially expressed lncRNAs, such as ATPase Na+/K+ transporting family member beta 4 (ATP1B4), and protein kinase AMP-activated alpha 1 catalytic subunit (PRKAA1), were enriched in TGF-β signaling pathway (oas04350), mTOR signaling pathway (oas04150), PI3K-Akt signaling pathway (oas04151), MAPK signaling pathway (oas04010) and Ras signaling pathway (oas04014), which were found to participate in hair follicle organogenesis and regeneration (Figure 4B, Table S7). Several differentially expressed lncRNAs were involved in hair follicle development, such as TCONS_00279168 and TCONS_00088322 enriched in PI3K-Akt, Ras signaling pathway and mTOR signaling pathway, respectively.

**Figure 5:**
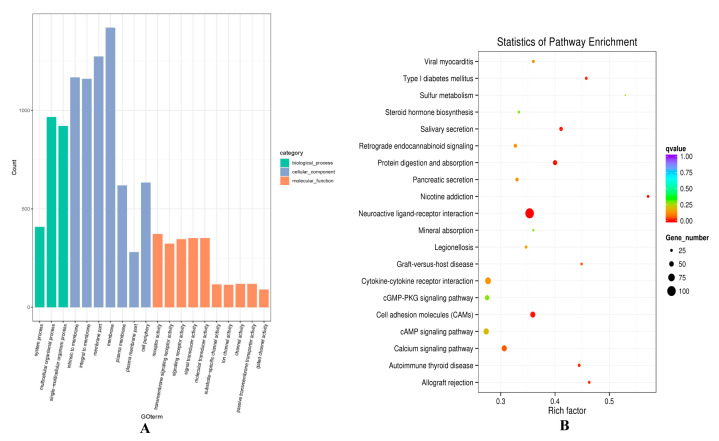
GO and KEGG analysis of differentially expressed lncRNAs target mRNA through co-expression analysis in hair follicle. (**A**) The enrichment GO terms between small waves and straight wool. (**B**) The enrichment pathways between small waves and straight wool.

Regarding the co-expression results, GO terms of the lncRNAs potential targets were enriched in some development process, such as system process (0003008), multicellular organismal process (GO:0032501), and single-multicellular organism process (GO:0044707) (Figure 5A, Table S8). KEGG enrichment analysis revealed that the target genes Fibroblast Growth Factor 12 (FGF12) and ATP1B4 of TCONS_00279168 were enriched in the PI3K-Akt signaling pathway (oas04151), MAPK signaling pathway (oas04010), and Ras signaling pathway (oas04014). There was also a very classical signaling pathway cAMP (oas04024) found, which was involved in calcium absorption to regulate Ca^2+^ concentrations and subsequent cell growth. (Figure 5B, Table S9). 

### 3.4. lncRNA-mRNA Relationship Analysis of Differentially Expressed lncRNAs and mRNAs

**Figure 6:**
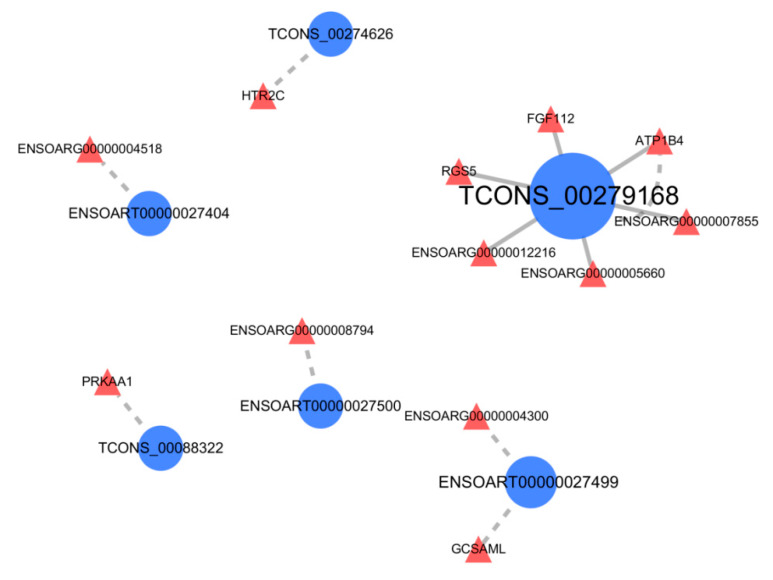
The result of the interaction relationship between differentially expressed lncRNAs and their corresponding differentially expressed mRNAs. Blue and red, circles, and triangles represent lncRNAs and mRNAs. The dotted line represents the co-location relationship, and the solid line represents the co-expression relationship. Furthermore, bigger circles indicate that these lncRNAs have stronger connections with the differentially expressed mRNAs.

In most cases, lncRNA can regulate gene expression to affect traits indirectly at the transcriptional or post-transcriptional level. To more accurately identify the lncRNAs that may participate in hair follicle morphogenesis, lncRNA-mRNA interaction relationship analysis of differentially expressed mRNAs and lncRNAs were constructed (Figure 6). A total of 6 differentially expressed lncRNAs were associated with 12 differentially expressed mRNAs, such as TCONS_00279168, TCONS_00088322, *PRKAA1*, *ATP1B4*, *FGF12.* Among the results, TCONS_00279168 targeted to *ATP1B4* and *FGF12,* and *ATP1B4* was also the source gene of TCONS_00279168. They were believed to have a close relationship. 

## 4. Discussion

The formation of Hu sheep lambskin is influenced by wool curvature and has a complex mechanism. Hair follicle growth and development is the basis of the formation of wool curvature. The mechanism of dermal papilla cell growth in the hair follicle is proved to be responsible for hair curvature formation through various signaling in both direct or indirect ways. Increasingly, researches have been reported on different regulators in hair follicle growth and development such as BMPs [11], FGFs [33], WNTs [8], and IGFs [34]. Directly, Wnt and mTOR signaling were the typical pathways to directly explain the molecular mechanism of hair curvature [4]. Indirectly, the North signaling, Wnt/Ca^2+^ signaling, MAPK signaling, and others, in some ways, can explain the formation of hair curvature [13]. However, the studies on non-coding RNAs, which were previously considered of unknown function, in hair follicle morphogenesis are still limited. Our study can path the way for the in-depth research on lncRNAs in relation to Hu sheep lambskin, as well a discovery of the feasible roles in hair follicle morphogenesis.

9954 lncRNAs were identified in this study, and most of the lncRNAs were concentrated in the length range of 200 to 1000 nt and had two or three exons, which consisted of the universal characteristic in lncRNA. Then, 75 differentially expressed lncRNAs were screened from hair follicles between small waves and straight wool of Hu sheep lambskin, which implied their extensive roles in the sheep hair follicle. In our previous study, 41 differentially expressed lncRNAs were detected between large and small waves, and several differentially expressed lncRNAs (TCONS_00015929 and TCONS_00010609) also existed in small waves and straight wool [35], which may play a role in hair follicle development. Obviously, more differentially expressed lncRNAs were found between small waves and straight wool groups. Several lncRNAs, such as TCONS_00279168, TCONS_00088322, were unique and significantly expressed between the small waves and straight wool groups, which did not appear in large and small waves groups. Collectively, we hypothesized that the greater difference between small waves and straight wool groups contributed to the results. 

After co-location, co-expression analysis, and functional annotation were performed to explore the biological role of the differentially expressed lncRNAs. Regarding KEGG analysis, we surprisingly found that the pathways named TGF-β, mTOR, MAPK, PI3K-Akt, and the Ras signaling pathway, were significantly enriched. In our previous work, the TGF-β, MAPK signaling pathway was also a significantly enriched pathway, but the MAPK, PI3K-Akt, and Ras signaling pathway did not achieve a significant level, which may also be due to the litter difference between small and large waves. Previous researches insight has been gained to reveal that the role of TGF-β and MAPK signaling pathways in hair follicle growth and development [4,36,37,38]. TGF-β signaling pathway was revealed to promote apoptosis and inhibit the proliferation of hair follicle cells from regulating hair follicle morphogenesis [39]. The regulators in the MAPK signaling pathway and other hairy-related genes translated into a cyclic wave of expression and serves as a positional cue for FGF signaling pathway (a vital regulatory factor that regulates the periodic changes of hair follicles) through Notch signaling pathway (key member in the differentiation and maturation of hair follicles) [33,40,41]. Especially the activated MAPK signaling could act as positive signaling to promote dermal papilla cell proliferation, which is an important factor for the formation of hair curvature [4,42]. In addition, mTOR signaling pathway is a regulator of dermal papilla cells leading to multiple papillary centers (MPC) formation to cause the phenotype of wool curvature [4]. PI3K-Akt and Ras signaling pathway, and their function should not be ignored. Based on the study on the positive effect of 12-o-tetradecanoylphorbol-13-acetate (*TPA*) promoting hair follicle regeneration, Qiu et al. [43] found TPA could activate the PI3K-Akt signaling pathway to accelerate hair follicle stem cells proliferation. More interestingly, activation of PI3K-Akt signaling in the hair follicle can increase the expression level of *TGF-β2*. *TGF-β2*, the most important signaling in TGF-β signaling pathway, not only promotes hair regeneration but also has an influence on hair curvature [4,44]. The Ras signaling pathway was identified to regulate cellular proliferation and differentiation, and *RAS* mutation could affect hair follicle morphogenesis. Ras signaling was considered a regular of FGF signaling, and even regulated hair follicle morphogenesis through fine-turning the expression level of Shh signal [45,46], it seems plausible that PI3K-Akt and Ras signaling pathways may be related to the follicle growth and development in sheep. 

According to the interaction network analysis, TCONS_00279168 and TCONS_00088322 are located in *ATP1B4* and *PRKAA1*, respectively. The expression level of TCONS_00279168 and *ATP1B4*, *FGF12* in SM and ST were consistent, and the correlation coefficient reached a significant level. Functional annotation showed that these lncRNAs and mRNAs were enriched in hair follicle development pathways, such as mTOR, MAPK, PI3K-Akt, and Ras signaling pathway. At present, there is no clear report that *PRKAA1* is involved in hair follicle development, but it plays an important role in cell proliferation, such as cancer and muscle cells [47,48]. Since *PRKAA1* was enriched in the mTOR pathway in this study, it is worth investigating whether *PRKAA1* and it co-located lncRNA TCONS_00088322 can affect the proliferation of hair follicle cells.

*FGF12* belongs to the FGF family [49], similar to other FGF members [50], FGF12 function as a secretory antagonist of signaling molecules such as Wnt, TGF [51,52]. FGF signaling was the epithelial signal that regulates the dermal condensate formation and subsequently affects hair follicle morphogenesis. *FGF20*, another gene of the FGF family, was confirmed to be downstream of Wnt and Edr signaling and can even affect the expression levels of some important genes, such as *bmp2*, *bmp4,* and *Edar* [39]. Although there is no evidence to prove that *FGF12* can directly regulate the growth and development of hair follicles, considering that it is one of the FGF signals, it is highly likely to play an important role in hair follicle morphogenesis. Coincidently, *FGF12* was also found enriched in the MAPK signaling pathway, combined with the differentially expressed analysis result, it can be speculated that TCONS_00279168 may involve in the transcriptional activation of *FGF12*, eventually regulates hair follicle growth and development indirectly through the MAPK signaling pathway, the specific functional roles of the lncRNAs in follicle growth and development affecting hair curvature in sheep require further careful characterization. 

Interestingly, according to the analysis of lncRNAs, the source gene of TCONS_00279168 was *ATP1B4*. Pearson correlation coefficients and P-value were calculated for target gene predictions. The co-expression networks showed that *ATP1B4* was also the target gene of TCONS_00279168. Therefore, *ATP1B4* is not only the source gene of TCONS_00279168 but also its target gene. *ATP1B4* is a member of ATPase Na+/K+ transporting family, which is believed to contribute to the activity of the MAPK signaling pathway, which is well supported by the studies in humans [53,54]. Furthermore, *ATP1B4*, as an important member of ATPase Na+/K+ transporting beta M family, which is involved in the regulation of gene expression level in TGF-β signaling pathway as exemplified by an increase of expression of inhibitory Smad7. As stated about the dominance of the TGF superfamily in hair follicle growth (inhibit the proliferation of hair follicles) [55], some undiscovered relationships between the *ATP1B4,* ATPase Na+/K+ transporting family, TCONS_00279168 and hair follicle growth presumably exist. Certainly, some other differentially expressed lncRNAs and mRNAs in the network are also worthy of an in-depth investigation of the relationship with hair follicle development.

## 5. Conclusions

In summary, 25 differentially expressed mRNAs and 75 differentially expressed lncRNAs were screened between small waves and straight wool, including 12 up-regulated, 13 down-regulated differentially expressed mRNAs, and 16 up-regulated, 59 down-regulated differentially expressed lncRNAs. We also revealed many meaningful lncRNAs and mRNAs through functional annotation analysis and interaction network method, such as TCONS_00279168, TCONS_00088322, *FGF12*, *ATP1B4*, *PRKAA1*. The results of our research can provide the basis for understanding the function and molecular regulation mechanism of the lncRNAs in hair follicle growth in sheep.

## Figures and Tables

**Table 1:** The information of primers.

Type	Gene ID/Symbol	Primer Sequences (5′–3′)		Product Length (bp)	Accession Number
lncRNAs	XLOC_051456	F	CACTCAGCCTTCTTCACAGT	158	
		R	AGACGCTTACTCCTTGGAAGA		
	XLOC_079348	F	TGCCATGATCTTTGTTTTCTGAA	193	
		R	GCATCAAGATTGCCGGGAG		
	XLOC_153474	F	TCCCTGAGTAGTGTGACAGC	181	
		R	GCCTTGACTAGACGGACCTT		
	XLOC_178815	F	TGGCAAAGTAATGTCTCTGCT	190	
		R	TCCGGTCCCATCACTTCATG		
	TCONS_00279168	F	ACTGAGTTCAGACCAGCAGC	131	
		R	TGTAACCGGATCAACGCCTC		
mRNAs	*SORCS3*	F	ACCAGTGGAATCGTGTGACC	186	XM_004020175.4
		R	AAGTTACTCGCCCCAGATGC		
	*SOBP*	F	AGAAATAAGGTATGTGACTGGTGT	85	XM_015097362.2
		R	ACTGAAGCCTTCTTTCCCCG		
	*GABRA1*	F	CCCATGCCTGCCCTCTAAAA	121	XM_004009041.4
		R	TGGTTTAGGCGTGACCCATC		
	*TMEFF2*	F	GGCTGGAACTGCTCTGGTTA	169	XM_027965049.1
		R	TCTCCCCATTGGAACCACAC		
	*FGF12*	F	TGAAGGCCAGCCTCTATGTG	160	FJ958355.1
		R	AACCAAGCTCGGCCTGATTC		
Reference gene	*GAPDH*	F	GTCGGAGTGAACGGATTTGG	196	HM043737.1
		R	CATTGATGACGAGCTTCCCG		

**Table 2:** The information of RNA-seq result data.

Sample	Raw Bases (G)	Raw Reads	Clean Bases (G)	Clean Reads	Error Rate (%)	GC Content (%)	Total Mapped (%)	Uniquely Mapped (%)
SM1	15.90	106,025,390	15.67	104,490,060	0.03	49.78	85.46	79.26
SM2	15.25	101,680,458	15.02	100,165,540	0.03	53.10	81.29	74.09
SM3	18.48	123,207,764	18.12	120,790,388	0.03	52.13	85.02	78.64
ST1	14.96	99,732,676	14.70	97,967,308	0.03	53.83	82.05	73.95
ST2	12.58	83,884,650	12.38	82,542,358	0.03	52.33	82.54	75.64
ST3	16.93	112,891,936	16.43	109,522,416	0.03	53.84	87.34	74.56

## Data Availability

The sequence data from this article has been submitted to SRA with the Accession No. SRR11793747, SRR11793748, SRR11793749, SRR11793750, SRR11793751, SRR11793752.

## Supplementary Materials

The following are available online at https://www.mdpi.com/2076-2615/10/6/1035/s1, Table S1: Accession number of lncRNAs for primer design, Table S2: List of differentially expressed lncRNAs between small waves and straight wool groups, Table S3: List of differentially expressed mRNAs between small waves and straight wool groups, Table S4: The co-location analysis of differentially expressed lncRNA, Table S5: The co-expression analysis of differentially expressed lncRNAs, Table S6: List of GO analysis of the co-location result, Table S7: List of KEGG analysis of co-location result, Table S8: List of GO analysis of the co-expression result, Table S9: List of KEGG analysis of co-expression result.

## References

[1] Gao W., Sun W., Yin J., Lv X., Bao J., Yu J., Wang L., Jin C., Hu L. (2017). Screening candidate microRNAs (miRNAs) in different lambskin hair follicles in Hu sheep. PLoS ONE.

[2] Lv X., Sun W., Yin J., Ni R., Su R., Wang Q., Gao W., Bao J., Yu J., Wang L. (2016). An Integrated Analysis of MicroRNA and mRNA Expression Profiles to Identify RNA Expression Signatures in Lambskin Hair Follicles in Hu Sheep. PLoS ONE.

[3] Sun W., Ni R., Yin J.F., Musa H.H., Ding T., Chen L. (2013). Genome array of hair follicle genes in lambskin with different patterns. PLoS ONE.

[4] Nissimov J.N., Chaudhuri A.B.D. (2014). Hair curvature: A natural dialectic and review. Boil. Rev..

[5] Yucel G., Van Arnam J., Means P.C., Huntzicker E., Altindag B., Lara M.F., Yuan J., Kuo C., Oro A.E. (2014). Partial proteasome inhibitors induce hair follicle growth by stabilizing beta-catenin. Stem Cells.

[6] Rile N., Liu Z., Gao L., Qi J., Zhao M., Xie Y., Su R., Zhang Y., Wang R., Li J. (2018). Expression of Vimentin in hair follicle growth cycle of inner Mongolian Cashmere goats. BMC Genom..

[7] Wang Q., Wang Y., Pang S., Zhou J., Cai J., Shang J. (2019). Alcohol extract from Vernonia anthelmintica willd (L.) seed counteracts stress-induced murine hair follicle growth inhibition. BMC Complement. Altern. Med..

[8] Tripurani S.K., Wang Y., Fan Y.X., Rahimi M., Wong L., Lee M.H., Starost M.F., Rubin J.S., Johnson G.R. (2018). Suppression of Wnt/beta-catenin signaling by EGF receptor is required for hair follicle development. Mol. Biol. Cell.

[9] Wu Z., Zhu Y., Liu H., Liu G., Li F. (2020). Wnt10b promotes hair follicles growth and dermal papilla cells proliferation via Wnt/beta-Catenin signaling pathway in Rex rabbits. Biosci. Rep..

[10] Calvo-Sanchez M.I., Fernandez-Martos S., Carrasco E., Moreno-Bueno G., Bernabeu C., Quintanilla M., Espada J. (2019). A role for the Tgf-beta/Bmp co-receptor Endoglin in the molecular oscillator that regulates the hair follicle cycle. J. Mol. Cell Biol..

[11] Genander M., Cook P.J., Ramskold D., Keyes B.E., Mertz A.F., Sandberg R., Fuchs E. (2014). BMP signaling and its pSMAD1/5 target genes differentially regulate hair follicle stem cell lineages. Cell Stem Cell.

[12] Pujades C., Kamaid A., Alsina B., Giraldez F. (2006). BMP-signaling regulates the generation of hair-cells. Dev. Biol..

[13] Kan L., Liu Y., McGuire T.L., Bonaguidi M.A., Kessler J.A. (2011). Inhibition of BMP signaling in P-Cadherin positive hair progenitor cells leads to trichofolliculoma-like hair follicle neoplasias. J. Biomed. Sci..

[14] Ohuchi H., Tao H., Ohata K., Itoh N., Ono K. (2003). Fibroblast growth factor 10 is required for proper development of the mouse whiskers. Biochem. Biophys. Res. Commun..

[15] Mimeault M., Batra S.K. (2013). Hypoxia-inducing factors as master regulators of stemness properties and altered metabolism of cancer- and metastasis-initiating cells. J. Cell. Mol. Med..

[16] Driskell R.R., Clavel C., Rendl M., Watt F.M. (2011). Hair follicle dermal papilla cells at a glance. J. Cell Sci..

[17] Christiano A.M. (2008). Hair follicle epithelial stem cells get their sox on. Cell Stem Cell.

[18] Wang J., Zhu S., Meng N., He Y., Lu R., Yan G.R. (2019). ncRNA-Encoded Peptides or Proteins and Cancer. Mol. Ther..

[19] Li J., Xue Y., Amin M.T., Yang Y., Yang J., Zhang W., Yang W., Niu X., Zhang H.Y., Gong J. (2020). ncRNA-eQTL: A database to systematically evaluate the effects of SNPs on non-coding RNA expression across cancer types. Nucleic Acids Res..

[20] Zhang G., Cai J. (2019). Evaluation of prognostic value of lncRNA BANCR in tumor patients: A systematic review and meta-analysis. J. Buon.

[21] Yue Y., Guo T., Yuan C., Liu J., Guo J., Feng R., Niu C., Sun X., Yang B. (2016). Integrated Analysis of the Roles of Long Noncoding RNA and Coding RNA Expression in Sheep (*Ovis aries*) Skin during Initiation of Secondary Hair Follicle. PLoS ONE.

[22] Si Y., Bai J., Wu J., Li Q., Mo Y., Fang R., Lai W. (2018). LncRNA PlncRNA1 regulates proliferation and differentiation of hair follicle stem cells through TGFbeta1mediated Wnt/betacatenin signal pathway. Mol. Med. Rep..

[23] Kong L., Zhang Y., Ye Z.Q., Liu X.Q., Zhao S.Q., Wei L., Gao G. (2007). CPC: Assess the protein-coding potential of transcripts using sequence features and support vector machine. Nucleic Acids Res..

[24] Sun L., Luo H., Bu D., Zhao G., Yu K., Zhang C., Liu Y., Chen R., Zhao Y. (2013). Utilizing sequence intrinsic composition to classify protein-coding and long non-coding transcripts. Nucleic Acids Res..

[25] Finn R.D., Bateman A., Clements J., Coggill P., Eberhardt R.Y., Eddy S.R., Heger A., Hetherington K., Holm L., Mistry J. (2014). Pfam: The Protein Families Database. Nucleic Acids Res..

[26] Li A., Zhang J., Zhou Z., Zhou Z.Y. (2014). PLEK: A tool for predicting long non-coding RNAs and messenger RNAs based on an improved k- mer scheme. BMC Bioinform..

[27] Trapnell C., Williams B.A., Pertea G., Mortazavi A., Kwan G., van Baren M.J., Salzberg S.L., Wold B.J., Pachter L. (2010). Transcript assembly and quantification by RNA-Seq reveals unannotated transcripts and isoform switching during cell differentiation. Nat. Biotechnol..

[28] Mak D.R., Davis J.M., Gordon K.S. (2010). edgeR: a Bioconductor package for differential expression analysis of digital gene expression data. Bioinformatics.

[29] Wang L.K., Feng Z.X., Wang X., Wang X.W., Zhang X.G. (2010). DEGseq: An R package for identifying differentially expressed genes from RNA-seq data. Bioinformatics.

[30] Demchak B., Hull T., Reich M., Liefeld T., Smoot M., Ideker T., Mesirov J.P. (2014). Cytoscape: The network visualization tool for GenomeSpace workflows. F1000Research.

[31] Nie Y.F., Li S.M., Zheng X.T., Chen W.S., Li X.E., Liu Z.W., Hu Y., Qiao H.S., Qi Q.Q., Pei Q.B. (2018). Transcriptome reveals long non-coding rnas and mrnas involved in primary wool follicle induction in carpet sheep fetal skin. Front. Physiol..

[32] Livak K.J., Schmittgen T.D. (2001). Analysis of relative gene expression data using real-time quantitative PCR and the 2(-delta delta c(t)) method. Methods.

[33] Lush M.E., Diaz D.C., Koenecke N., Baek S., Boldt H., St P.M., Gaitan-Escudero T., Romero-Carvajal A., Busch-Nentwich E.M., Perera A.G. (2019). scRNA-Seq reveals distinct stem cell populations that drive hair cell regeneration after loss of Fgf and Notch signaling. eLife.

[34] Ahn S.Y., Pi L.Q., Hwang S.T., Lee W.S. (2012). Effect of IGF-I on Hair Growth Is Related to the Anti-Apoptotic Effect of IGF-I and Up-Regulation of PDGF-A and PDGF-B. Ann. Dermatol..

[35] Lv X.Y., Gao W., Jin C.Y., Wang Y., Chen W.H., Wang L.H., Zou S.X., Sheng S.X., Chen L., Sun W. (2019). Divergently expressed rna identification and interaction prediction of long non-coding rna and mrna involved in hu sheep hair follicle. Sci. Rep..

[36] Akilli O.O., Pakula H., Chmielowiec J., Qi J., Stein S., Lan L., Sasaki Y., Rajewsky K., Birchmeier W. (2015). Gab1 and Mapk Signaling Are Essential in the Hair Cycle and Hair Follicle Stem Cell Quiescence. Cell Rep..

[37] Sohn K.M., Jeong K.H., Kim J.E., Park Y.M., Kang H. (2015). Hair growth-promotion effects of different alternating current parameter settings are mediated by the activation of Wnt/beta-catenin and MAPK pathway. Exp. Dermatol..

[38] Liu J., Xu Y., Wu Q., Ding Q., Fan W. (2018). Sirtuin1 protects hair follicle stem cells from TNFalpha-mediated inflammatory stress via activating the MAPK-ERK-Mfn2 pathway. Life Sci..

[39] Xenakis N., Mok K.W., Rendl M. (2019). An updated classification of hair follicle morphogenesis. Exp. Dermatol..

[40] Sawada A., Shinya M., Jiang Y.J., Kawakami A., Kuroiwa A., Takeda H. (2001). Fgf/MAPK signaling is a crucial positional cue in somite boundary formation. Development.

[41] Jiang L., Xu J., Jin R., Bai H., Zhang M., Yang S., Zhang X., Zhang X., Han Z., Zeng S. (2018). Transcriptomic analysis of chicken cochleae after gentamicin damage and the involvement of four signaling pathways (Notch; FGF.; Wnt and BMP) in hair cell regeneration. Hear. Res..

[42] Xiao S.N., Wang J., Chen Q., Miao Y., Hu Z.Q. (2019). The mechanism of activated platelet-rich plasma supernatant promotion of hair growth by cultured dermal papilla cells. J. Cosmet. Dermatol..

[43] Qiu W.M., Lei M.X., Zhou L., Bai X.F., Lai X.D., Yu Y., Yang T., Lian X.H. (2017). Hair follicle stem cell proliferation, akt and wnt signaling activation in tpa-induced hair regeneration. Histochem. Cell Boil..

[44] Zhu H.L., Gao Y.H., Yang J.Q., Li J.B., Gao J. (2018). Serenoa repens extracts promote hair regeneration and repair of hair loss mouse models by activating TGF-β and mitochondrial signaling pathway. Eur. Rev. Med. Pharmacol. Sci..

[45] Mukhopadhyay A., Krishnaswami S.R., Cowing-Zitron C., Hung N.J., Reilly-Rhoten H., Burns J., Yu B.D. (2013). Negative regulation of Shh levels by Kras and Fgfr2 during hair follicle development. Dev. Biol..

[46] Rishikaysh P., Dev K., Diaz D., Qureshi W.M.S., Filip S., Mokry J. (2004). Signaling involved in hair follicle morphogenesis and development. Int. J. Mol. Sci..

[47] Zhang Y.M., Zhou X.C., Cheng L., Wang X., Zhang Q.L., Zhang Y.W., Sun S.Y. (2020). PRKAA1 promotes proliferation and inhibits apoptosis of gastric cancer cells through activating JNK1 and Akt pathways. Oncol. Res..

[48] Gui D., Cui Z.M., Zhang L., Yu C., Yao D., Xu M., Chen M.Y., Wu P.L., Li G.P., Wang L.X. (2017). Salidroside attenuates hypoxia-induced pulmonary arterial smooth muscle cell proliferation and apoptosis resistance by upregulating autophagy through the AMPK-mTOR-ULK1 pathway. BMC Pulm. Med..

[49] Nakayama F., Yasuda T., Umeda S., Asada M., Imamura T., Meineke V., Akashi M. (2011). Fibroblast growth factor-12 (FGF12) translocation into intestinal epithelial cells is dependent on a novel cell-penetrating peptide domain: Involvement of internalization in the in vivo role of exogenous FGF12. J. Biol. Chem..

[50] Al-Mehmadi S., Splitt M., Ramesh V., DeBrosse S., Dessoffy K., Xia F., Yang Y., Rosenfeld J.A., Cossette P., Michaud J.L. (2016). FHF1 (FGF12) epileptic encephalopathy. Neurol. Genet..

[51] Mossahebi-Mohammadi M., Quan M., Zhang J.S., Li X. (2020). FGF Signaling Pathway: A Key Regulator of Stem Cell Pluripotency. Front. Cell Dev. Boil..

[52] Hillege M., Galli C.R., Offringa C., de Wit G., Jaspers R.T., Hoogaars W. (2020). TGF-beta Regulates Collagen Type I Expression in Myoblasts and Myotubes via Transient Ctgf and Fgf-2 Expression. Cells-Basel.

[53] Neves L., Goncalves E., Cavalli J., Vieira G., Laurindo L.R., Simoes R.R., Coelho I.S., Santos A., Marcolino A.M., Cola M. (2018). Photobiomodulation Therapy Improves Acute Inflammatory Response in Mice: The Role of Cannabinoid Receptors/ATP-Sensitive K(+) Channel/p38-MAPK signaling Pathway. Mol. Neurobiol..

[54] Guo W., Ma J., Yang Y., Guo S., Zhang W., Zhao T., Yi X., Wang H., Wang S., Liu Y. (2020). ATP-Citrate Lyase Epigenetically Potentiates Oxidative Phosphorylation to Promote Melanoma Growth and Adaptive Resistance to MAPK Inhibition. Clin. Cancer Res..

[55] Korneenko T.V., Pestov N.B., Ahmad N., Okkelman I.A., Dmitriev R.I., Shakhparonov M.I., Modyanov N.N. (2016). Evolutionary diversification of the BetaM interactome acquired through co-option of the ATP1B4 gene in placental mammals. Sci. Rep..

